# A Color-Based Multispectral Imaging Approach for a Human Detection Camera

**DOI:** 10.3390/jimaging11040093

**Published:** 2025-03-21

**Authors:** Shuji Ono

**Affiliations:** Fujifilm Corporation, Kaisei 250-8577, Ashigara-kami, Kanagawa, Japan; shuji.ono@fujifilm.com or shuji.ono.1960@gmail.com

**Keywords:** color imaging, multispectral imaging, human detection, machine learning, real-time processing, spectral reflectance

## Abstract

In this study, we propose a color-based multispectral approach using four selected wavelengths (453, 556, 668, and 708 nm) from the visible to near-infrared range to separate clothing from the background. Our goal is to develop a human detection camera that supports real-time processing, particularly under daytime conditions and for common fabrics. While conventional deep learning methods can detect humans accurately, they often require large computational resources and struggle with partially occluded objects. In contrast, we treat clothing detection as a proxy for human detection and construct a lightweight machine learning model (multi-layer perceptron) based on these four wavelengths. Without relying on full spectral data, this method achieves an accuracy of 0.95, precision of 0.97, recall of 0.93, and an F1-score of 0.95. Because our color-driven detection relies on pixel-wise spectral reflectance rather than spatial patterns, it remains computationally efficient. A simple four-band camera configuration could thus facilitate real-time human detection. Potential applications include pedestrian detection in autonomous driving, security surveillance, and disaster victim searches.

## 1. Background and Objectives

### 1.1. Background and Significance

Human detection technologies are important for such applications as autonomous driving, security surveillance, and disaster rescue. At disaster sites, it is crucial to locate victims as quickly as possible. In autonomous driving, pedestrians must be detected accurately. Leveraging spectral characteristics may make it possible to achieve high accuracy with reduced computational load.

In this research, we developed a new human detection approach that uses the spectral (color) properties of clothing. Assuming outdoor daytime conditions and common fibers such as cotton, polyester, and wool, we hypothesize that fibers and dyes exhibit high reflectance in the near-infrared region, which can be exploited to detect people’s clothing with high accuracy. This limitation of fiber types is based on data indicating that cotton, polyester, and wool account for 79.9% of major global fibers [1].

To verify this hypothesis, the aim of this study was to show that by effectively and simply selecting wavelengths from the visible to near-infrared range, clothing and background can be distinguished. This work serves as an initial study toward realizing a “human detection camera” that can operate in real time.

### 1.2. Related Previous Studies

#### 1.2.1. Deep Learning-Based Human Detection

In recent years, the field of object detection has witnessed a rapid expansion of methods utilizing deep learning. This trend has led to significant improvements in the accuracy of human detection. Representative frameworks include the R-CNN series, which employs region proposals [2,3], the You Only Look Once (YOLO) series, which processes the entire image in a single pass [4], and the Single Shot MultiBox Detector (SSD) series, which leverages multi-scale features [5].

##### R-CNN Series (R-CNN, Fast R-CNN, Faster R-CNN, etc.)

The R-CNN series first generates multiple region proposals from the input image and then applies a convolutional neural network (CNN) within each proposed region to perform classification and bounding box regression. Through this procedure, relatively high accuracy can be achieved even for small objects or scenes with complex backgrounds. However, the dependence on computational resources—such as GPUs—and the limited inference speed mean that optimization is required for real-time inference or low-power applications.

##### YOLO Series

In the YOLO series, the entire image is fed into a CNN at once, predicting multiple bounding boxes and classes simultaneously in an end-to-end manner. This approach more readily achieves fast inference and has a computationally efficient implementation, making it suitable for real-time applications in many cases. On the other hand, additional refinements may be necessary in scenarios where small objects are densely packed or extremely high accuracy is demanded.

##### Single Shot MultiBox Detector (SSD) Series

The SSD approach detects objects by employing multiple default (anchor) boxes at different scales of feature maps processed in parallel. Because of its multi-scale adaptability, SSD is considered to strike a good balance between accuracy and speed. However, when the backbone network has a heavy computational load, inference time may increase, prompting a need for additional strategies such as model compression or knowledge distillation in embedded or small-device environments.

These deep-learning-based methods have achieved very high detection accuracy, supported by extensive datasets and the availability of GPU-based computation [6,7]. Nonetheless, operating large-scale models often requires significant computational power and energy consumption. Furthermore, if the deployment environment, lighting, or occlusion conditions differ from those in the training data, additional training or fine-tuning may be required to maintain robust performance. Consequently, in areas such as search-and-rescue, disaster response, and embedded systems, ongoing efforts focus on exploring the trade-off between detection performance and computational resources, as well as on model optimization and lightweight design.

#### 1.2.2. Human Detection Using Visible-Light Cameras, Thermography, and Spectral Information

While deep learning methods continue to drive higher detection accuracy, research that exploits wavelength regions other than visible light has also become increasingly active. Such efforts aim to ensure stable detection performance in nighttime or low-light conditions and other complex environments. The following sections introduce representative cases by categorizing them into (1) methods using only visible-light cameras, (2) methods using thermography (thermal imaging), and (3) methods using near-infrared or spectral information.

##### Human Detection Using Visible-Light Cameras

Prior to the emergence of deep learning, human detection methods such as the Histograms of Oriented Gradients (HOG) approach proposed by Dalal and Triggs [8] and the Deformable Parts Model (DPM) proposed by Felzenszwalb et al. [9] were widely employed. These techniques not only demonstrated relatively high accuracy but could also be implemented at near-real-time speeds, resulting in relatively low barriers to practical adoption. However, visible-light cameras are known to perform poorly under nighttime or low-light conditions, strong backlighting, or large variations in illumination, and detection errors tend to increase in scenes with complex backgrounds. In addition, they have inherent limitations in dealing with occlusion and the wide variety of clothing. After deep learning gained prominence, models trained on large-scale datasets such as COCO or PASCAL VOC became mainstream. Nevertheless, even these models can suffer performance degradation under conditions such as nighttime or inclement weather.

##### Human Detection Using Thermography (Thermal Imaging)

Thermal imaging techniques utilize the temperature difference between humans and their surroundings, making it easier to detect humans under poor lighting or nighttime conditions. In fact, large-scale datasets that combine visible-light and thermal images—such as the KAIST Multispectral Pedestrian Detection Benchmark [10] and the CVC-14 dataset [11]—have been developed, and various deep-learning-based methods have been actively studied and compared using these data [12]. While this approach generally exhibits strong detection capability at night or in dark areas, and is relatively unaffected by background clutter, it also faces practical challenges. These challenges include the higher cost and often lower resolution of thermal cameras, as well as the need for alignment (correcting viewpoint differences) when combining thermal images with visible-light images. Additionally, temperature distributions are affected by environmental conditions, leading to potential false detections and reduced accuracy.

##### Human Detection Using Near-Infrared and Spectral Information

In addition to visible-light and thermal imaging, research has also been reported on the use of near-infrared (NIR) or short-wavelength infrared (SWIR)—which occupy the intermediate range between visible and thermal wavelengths—or on employing hyperspectral cameras capable of fine-grained spectral measurements across multiple wavelength bands. Because human skin exhibits characteristic reflectance and absorption in certain wavelength ranges, such information can complement what cannot be captured by visible-light imaging alone [13]. The advantages of near-infrared or spectral methods include the ability to separate human subjects from the background by leveraging reflectance properties different from those in the visible spectrum and the possibility of effectively using thresholding or lightweight machine learning approaches without necessarily relying on large-scale deep learning. Another advantage is that one may utilize differences in environmental lighting or in the materials of objects, potentially enabling use cases in both daytime and nighttime scenarios (for example, combining near-infrared illumination with suitable camera sensors).

On the other hand, hyperspectral and multispectral devices are often more expensive and larger than conventional visible-light cameras, and the amount of data they generate can be substantial. Furthermore, selecting appropriate wavelength bands and determining the spectral resolution directly influence system cost, and achieving real-time processing becomes more difficult as the number of wavelength bands increases, due to lower frame rates and heavier computational demands.

### 1.3. Positioning of This Study

In this study, we focus on the observation that clothing often exhibits relatively high reflectance in the near-infrared range [14], and we propose a model that identifies clothing using only a small number of wavelength bands (four in total) between the visible and near-infrared domains. In general, clothing tends to occupy a larger area than human skin, and its spectral characteristics often differ from those of background objects. We therefore consider that it may be possible to detect human subjects without incurring the high computational costs typical of deep learning methods.

In this paper, we target outdoor daytime conditions and select an appropriate set of four wavelength bands based on hyperspectral data. We suggest that in the future, a simple camera design using a silicon CMOS image sensor with around four bands may be sufficient to achieve adequate clothing detection performance.

The academic contributions of this research can be summarized as follows:

- High-accuracy human detection using four wavelengths
  By selecting only four (narrow) wavelengths in an optimal combination, clothing can be accurately identified without using the entire visible to near-infrared range.
- Combining dimensionality reduction with random exploration
  Inspired by the successive halving strategy [15,16], which concentrates resources on promising candidates, we developed an exploratory method to reduce the number of wavelengths efficiently while maintaining high accuracy.
- Real-time suitability and simple configuration
  Because this method relies on spectral information on a per-pixel basis rather than on spatial patterns, it can offer fast inference and low memory usage. Compared with conventional deep learning methods, there is potential for drastically reduced computational resources. Because we prioritize real-time processing, we employ a pixel-wise method without image segmentation or super-pixel approaches, which typically increase computational complexity.
- Differences from existing methods and academic significance
  The existing approaches based on deep learning for object detection, or methods leveraging SWIR/thermal cameras effective for nighttime detection, have each demonstrated high performance. In contrast, our study could develop a new framework that achieves high accuracy for daytime outdoor clothing detection with minimal sensor and computational costs by using only four wavelengths in the visible–near-infrared range. This addresses low-cost and low-load scenarios not well covered by existing methods, thereby expanding the range of options for future human detection technologies.

### 1.4. Structure of This Paper

Section 2 describes the spectral characteristics of clothing. Section 3 explains the approach for distinguishing clothing from the background using machine learning, including model selection, the multi-layer perceptron (MLP) workflow, and training conditions. Section 4 presents classification experiments using the MLP model and discusses three stages: “full hyperspectral model”, “dimensionality reduction”, and “reduction in the number of bands”. Section 5 shows the verification results using actual images, and Section 6 provides discussion.

## 2. Spectral Characteristics of Clothing and Outdoor Background

### 2.1. Hyperspectral Camera Imaging and Terminology

To analyze the spectral reflectance of clothing against a background (inorganic materials and plants), we collected image data using a hyperspectral camera (Specim IQ by DHT Corporation, Kawasaki, Japan). This camera is equipped with a CMOS sensor covering 400–1000 nm, featuring 204 bands, a spectral resolution of 7 nm, a spatial resolution of 512 × 512 pixels, and an 8-bit intensity resolution.

We conducted daytime photography under stable illumination, requiring about 10–30 s per capture. Because the camera has limited sensitivity, we restricted this study to still-image data recorded outdoors in daylight; application to nighttime or indoor environments is left for future work.

A white reference board (provided with the camera) was used for calibration, and the Savitzky–Golay method [17] was applied to reduce noise in the acquired spectral data. As data at wavelengths above 900 nm contained a lot of noise, we used only the 40–900 nm range (167 bands) in this study.

In future implementations, our system will employ a four-band camera instead of a scanning hyperspectral camera, thereby reducing the acquisition time to near real-time levels.

In this paper, “spectral reflectance” (hereafter referred to as reflectance) refers to the wavelength-dependent characteristics of light that are either reflected or emitted from a target object when illuminated by broadband light sources, such as sunlight. This definition encompasses both conventional reflectance—the ratio of reflected light to incident light—and fluorescence effects, wherein absorbed energy is re-emitted at longer wavelengths. The spectral range considered in this study spans the visible and near-infrared (NIR) regions. In our experiments, the method using 167 bands between 400 and 900 nm is referred to as “hyperspectral”, whereas the approach that subsequently selects about 4–12 discrete wavelengths is called “multispectral”. Furthermore, an “optimal wavelength set” (OWS) denotes a combination of wavelengths that maximizes clothing-detection performance. In this study, we explored sets of approximately four or five bands that exhibited high performance and used them to distinguish clothing from the background.

### 2.2. Imaging Targets

**Figure 1:**
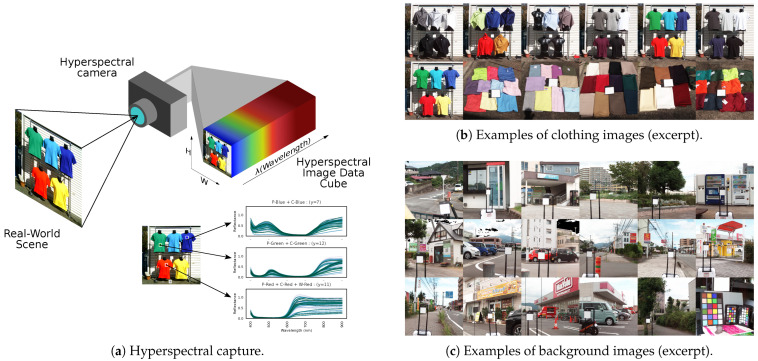
Hyperspectral imaging of clothing and scenery. (**a**) Hyperspectral capture. (**b**) Examples of clothing images (excerpt). (**c**) Examples of background images (excerpt).

We took hyperspectral images of 85 types of clothing and 35 cityscape scenes (Figure 1). (Figure 1b,c show examples of pseudo-color images generated by combining the bands for 650 nm, 530 nm, and 470 nm as RGB (Henceforth, pseudo-color images are shown by combining these three bands.)

#### 2.2.1. Hyperspectral Images of Clothing

We prepared various garments and fabrics made of cotton, polyester, and wool. These items were dyed with a range of colored dyes. The images of clothing in Figure 1b are representative pseudo-color shots of the captured data.

#### 2.2.2. Hyperspectral Images of the Background

As the background, we captured city and suburban landscapes, including roads, grass, wood, concrete, buildings, and vehicles in scenes with both sunny and cloudy daylight conditions. Figure 1c shows some examples of these background images in pseudo-color. The objects in the background appear to the human eye as green, gray, brown, and similar colors.

### 2.3. Observing the Spectra of Clothing and Outdoor Scenes

After imaging, we manually labeled target regions to produce label images. Clothing with similar spectral properties was consolidated into 39 labels, while the background was split into two categories—inorganic and plant. To accommodate the wide spectral variations exhibited by various types of clothing, the dataset was partitioned into multiple clothing classes, which are expected to enhance classification accuracy compared to using a single “clothing” label. Table 1 lists all 41 labels (label name, index number, and R-channel brightness in the labeled image).

**Figure 2:**
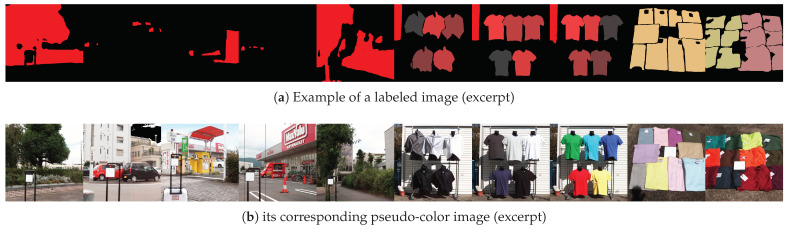
Example of a labeled image (**a**) and its corresponding pseudo-color (an artificially colored visualization) image (**b**).

Figure 2 shows an example of a labeled image alongside its corresponding pseudo-color image. In the labeled image, the R-channel brightness indicates the label class. For instance, plants are labeled with R = 255, and inorganic background is R = 0. Clothing has 39 distinct R brightness values depending on the fiber and color.

**Figure 3:**
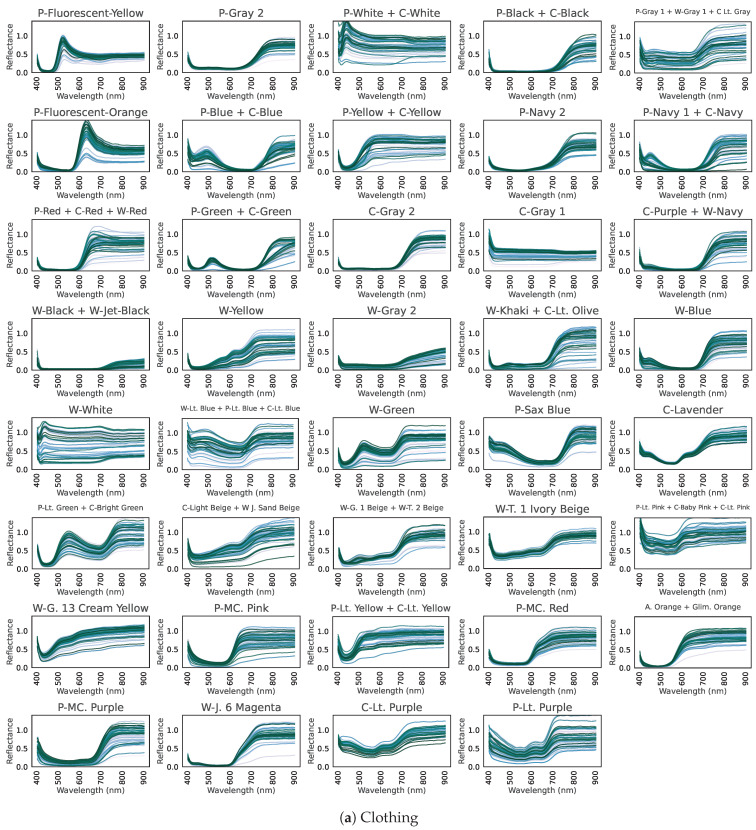

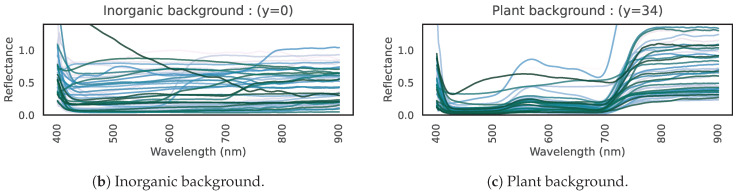
Hyperspectral reflectance curves, showing reflectance (vertical axis) over the visible–near-infrared range (horizontal axis) for 100 samples. (**a**) Clothing. (**b**) Inorganic background. (**c**) Plant background.

Figure 3 presents the hyperspectral reflectance curves for (a) 39 clothing labels, (b) an inorganic background, and (c) plants.

- In (a) for clothing, dyes absorb light at various visible wavelengths, creating the garment’s color. Meanwhile, nearly all clothing exhibits high reflectance in the near-infrared region, invisible to the human eye.
- In (b), inorganic objects do not show major changes across the visible to near-infrared range; there is almost no tendency for reflectance to rise in the near-infrared region.
- In (c), plants absorb blue light (~450 nm) and red light (~680 nm) while reflecting green (~550 nm), and they have high reflectance in the near-infrared. This is a well-known property of plant hyperspectral reflectance [18].

Thus, clothing and plants both exhibit high reflectance in the near-infrared, suggesting that distinguishing clothing from vegetation based on spectral information alone could be challenging.

**Figure 4:**
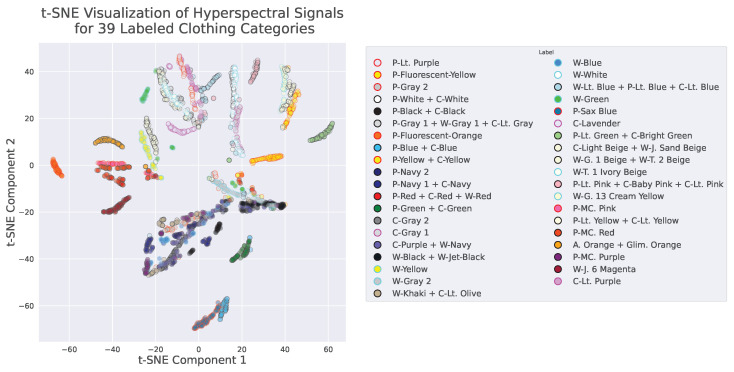
Distribution of the hyperspectral signals from clothing labeled into 39 categories. The data were analyzed using t-SNE, and circles are plotted at positions corresponding to the first and second components.

We performed a two-dimensional visualization of the hyperspectral signals from clothing labeled into 39 categories using t-SNE, as shown in Figure 4. Each plotted point (circle) in the figure is colored according to the clothing’s “visible color”.

## 3. Classification of Clothing and Background Using Machine Learning

### 3.1. Choice of Machine Learning Model

We employed machine learning to perform classification. To handle the variety of clothing, we adopted a multi-label classification model. Using scikit-learn [19], we compared five machine learning models—radial basis function support vector machine (RBF-SVM), random forest, gradient boosting, adaptive boosting, and multi-layer perceptron (MLP) [20,21,22,23,24]. As shown in Table 2, RBF-SVM and MLP gave the highest scores. Taking computational time into account, we ultimately selected the MLP.

In our preliminary experiments to determine the MLP structure using PyTorch Lightning (version 1.9.0), we varied the number of units in the hidden layers (testing sizes of 6, 10, 16, 32, and 64) while temporarily fixing the input layer to 20 units. We found that using two hidden layers with 16 units each resulted in performance that nearly saturated, while keeping computational cost and memory usage minimal (Table 3). Based on these results, we adopted an architecture comprising two hidden layers of 16 units each and an output layer of 49 units, thereby fixing the hidden and output layer configuration. However, in further experiments we plan to vary only the number of input layer units—while maintaining this optimized configuration—to investigate its impact. (Initially, the PyTorch implementation defined 49 output units; however, after consolidating the clothing labels into 39 categories, there are actually 39 clothing labels plus 2 background labels (i.e., 41 total). Since the unused output units had little effect on the training results, we continued using the 49-unit output design in our experiments.)

### 3.2. MLP Processing Flow and Training Conditions

**Figure 5:**
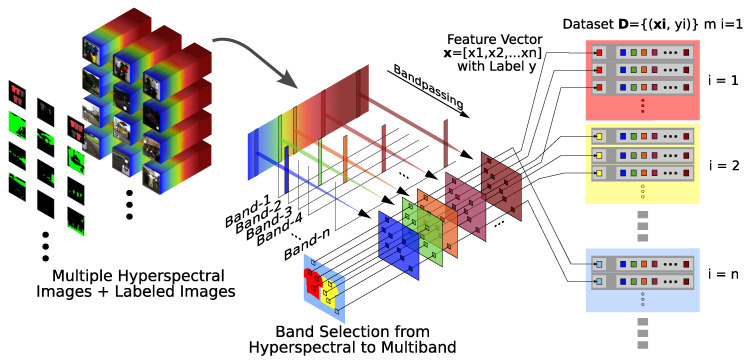
Multi-layer perceptron dataset creation workflow. Different colors and numbering indicate distinct label values in the dataset.

Figure 5 illustrates the process of creating the dataset for the MLP. The main steps are as follows:

1. Determine the number of wavelengths to use and choose which wavelengths to include, forming an OWS.
2. Extract the band images according to this wavelength set.
3. Randomly sample pixels from the labeled regions, taking 50 pixels per label for training.
4. Pair each pixel’s intensity vector with the corresponding label.

**Figure 6:**
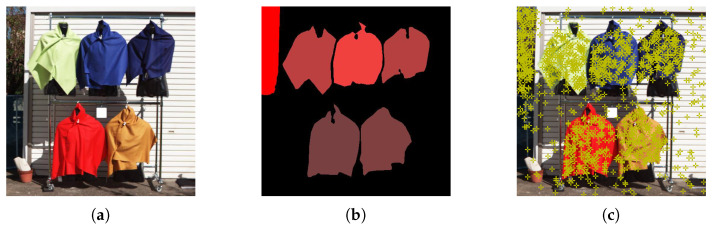
Sampling example, with moss-green dots indicating chosen pixels. (**a**) A pseudo-color image with 470, 580, 640 nm. (**b**) A labeled image. (**c**) A sampling example.

Figure 6 shows an example of sampled pixels (marked in dark green). To avoid mixing classes near label boundaries, we excluded pixels near those borders. Because only a sparse fraction of all pixels was extracted, overlap between training and test data was minimized.

Using PyTorch Lightning, we designed an MLP model that takes as input a feature vector with dimensions equal to the number of bands, passes it through two hidden layers (16 nodes each) with ReLU activation, and outputs a 49-dimensional (effectively 41-class) vector. We set the optimizer to Adam (lr = 0.001), the maximum epochs to 500, and EarlyStopping with a patience of 2. During inference, we applied argmax to the output score vector to select the class with the highest score. This yielded predictions for a single-label classification task.

Because the 35 images of city streets alone did not provide enough artificial-object samples, we augmented the inorganic background training data using color chart images (artificial colors). We also performed data augmentation in the brightness direction, scaling pixel intensities by factors of 0.68, 0.75, 0.83, 0.91, 1.00, 1.10, 1.21, 1.33, and 1.46 for nine stages. We balanced the total number of pixels in the inorganic + plant categories against the total number of clothing pixels. For each run, we performed training on about ~73,000 pixels and testing on about ~56,000 pixels. On an Intel i9-10900X/3.70-GHz CPU and two NVIDIA TITAN-V GPUs in parallel, each training run took roughly ~20 s.

## 4. Classification Experiments Using the MLP

### 4.1. Hyperspectral Model (167 Bands)

We first built an MLP model (MLP-167), taking as input 167 hyperspectral bands (400–900 nm). We computed a multi-label confusion matrix, then grouped all clothing classes as “P” (positive) and all background classes as “N” (negative). If two predicted labels belonged to clothing but differ in sub-label, we regarded that as a true positive. Similarly, any mismatch among background sub-labels was treated as a true negative. From this 2 × 2 confusion matrix, we derived the evaluation metrics accuracy, precision, recall, and F1-score (Because this is pixel-level classification, we used these metrics rather than IoU or mAP).

**Figure 7:**
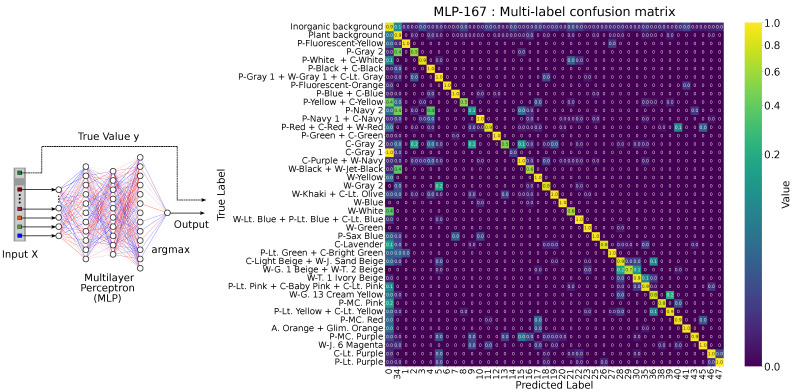
Multi-layer perceptron workflow and multi-label confusion matrix for 167 bands. The red and blue lines in the schematic represent, respectively, the relative magnitude and sign (positive in blue, negative in red) of the connection weights. These lines are illustrative only and do not reflect actual numerical weight values.

**Figure 8:**
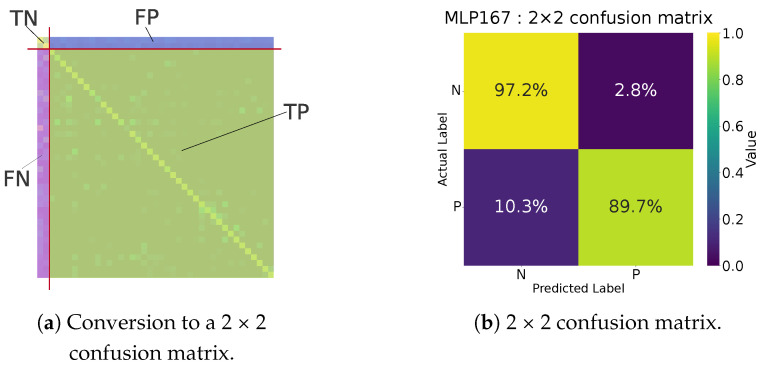
(**a**) Conversion to the clothing versus background matrix. (**b**) Example of the 2 × 2 confusion matrix for the 167-band model.

Figure 7 shows the MLP and the multi-label confusion matrix for 167 bands. Figure 8 shows (a) how we convert it to a 2 × 2 format and (b) the resulting 2 × 2 confusion matrix for the 167-band model. Because the MLP exhibits randomness in training and testing, performance varies somewhat each time. To compute the evaluation metrics, we trained the network at least 40 times with different random seeds and averaged the results (and did so likewise in subsequent experiments).

Table 4 lists the evaluation metrics for MLP-167, which are accuracy of 0.934, precision of 0.980, recall of 0.897, and F1 Score of 0.936. The respective standard deviations across 40 runs were 0.009, 0.004, 0.020, and 0.009, respectively. MLP-167 tended to miss some clothing (low recall, producing false negatives), likely due to the “curse of dimensionality” [25].

### 4.2. Improvement by Dimensionality Reduction

To mitigate the curse of dimensionality, we tested two approaches:

(1) Subsampling the wavelength axis at uniform intervals and
(2) Applying principal component analysis (PCA) to reduce the number of dimensions.

Table 5 compares the performance of MLP models with fewer bands using each approach. PCA did not substantially boost recall, but a 12-band multi-layer perceptron (MLP-12) covering 430~770 nm achieved accuracy of 0.95, precision of 0.97, recall of 0.93, and F1-score of 0.95.

**Figure 9:**
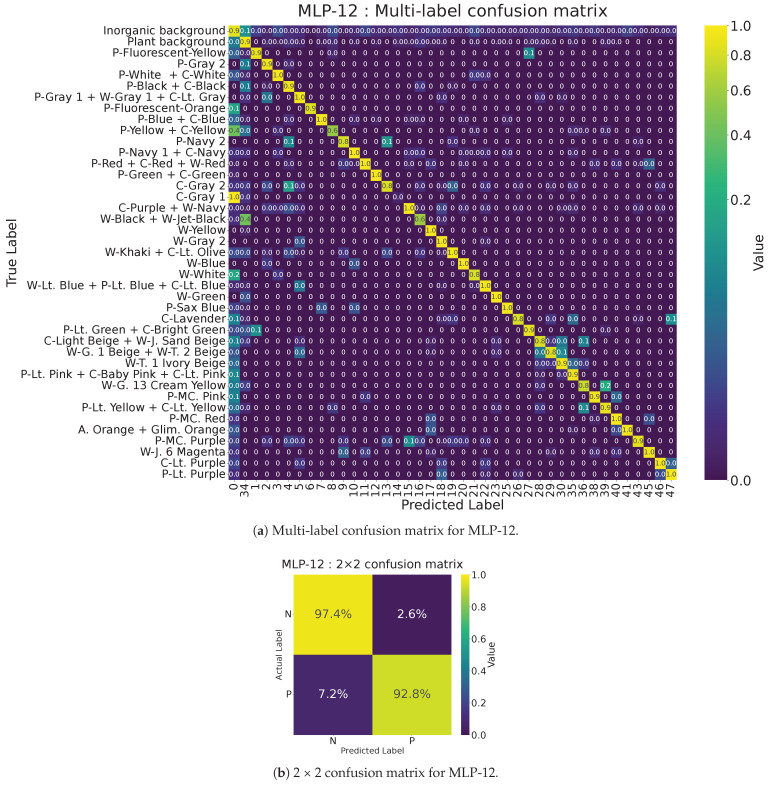
(**a**) Multi-label confusion matrix for 12-band multi-layer perceptron (MLP-12). (**b**) 2 × 2 confusion matrix for MLP-12.

Figure 9 shows (a) the multi-label confusion matrix for multi-layer perceptron with 12 bands (MLP-12) and (b) its 2 × 2 format. Compared with multi-layer perceptron with 167 bands, MLP-12 improved the recall score (false negatives decreased from 10.3% to 7.2%). This suggests that these metrics can serve as an approximate measure of how well an MLP model distinguishes clothing from the background using only spectral data.

### 4.3. Further Reduction in the Number of Bands

Even a 12-band system can be quite complex for a multispectral camera. We therefore explored whether the band count could be reduced further.

In the field of remote sensing, many wavelength selection techniques have been proposed for discriminating land surface materials such as crops and geology [26]. However, remote sensing typically deals with broad wavelength ranges, including SWIR to mid-infrared, for large-scale scene analysis, whereas our research focused on clothing detection in the visible–near-infrared range at relatively small spatial scales. Hence, directly applying existing wavelength-selection methods is difficult.

Drawing from the existing optimization algorithms, we devised a method to explore an optimal set of three to five bands.

#### 4.3.1. Relationship Between Band Count and Performance

**Figure 10:**
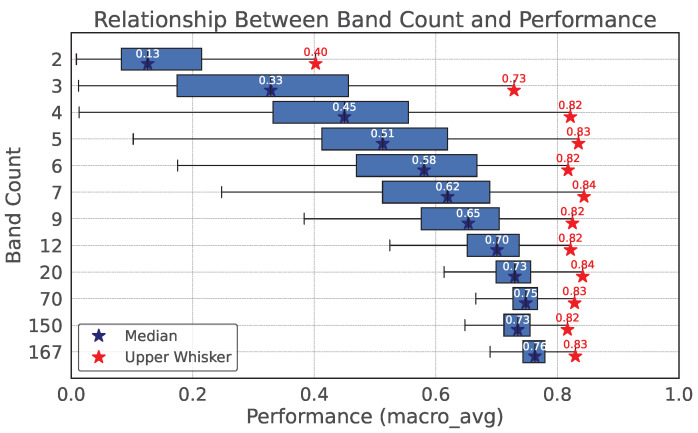
Relationship between band count and macro_avg.

We investigated how performance relates to the number of bands. Specifically, for each band count from 2 to 167, we generated 7000 random wavelength sets, trained an MLP, and recorded all macro_avg values. Here, macro_avg refers to the macro-averaged metric provided by scikit-learn’s evaluation module. It computes the unweighted mean of the per-class scores, meaning that each class contributes equally regardless of its sample count. Figure 10 shows the relationship between band count and classification performance (macro_avg).

As the band count decreased, the average performance dropped. However, the best performance at four bands was still high, implying the existence of a high-performing four-band wavelength set. Nonetheless, with only 7000 random trials, a high score might occur by chance given the randomness of the MLP.

#### 4.3.2. Searching for an Optimal Wavelength Set

From the above results, we focused on 4-band configurations while also exploring 5-band and 3-band OWSs. Inspired by established stagewise optimization methods such as successive halving [15,16,27,28], we combined random search with stepwise narrowing to develop an OWS search strategy. Although not a direct implementation of successive halving or other metaheuristics, the approach adopts their principle of efficiently pruning the search space to improve performance.

Concretely, the iterative search follows five steps:

1. Initial Candidate Generation Determine how many bands to explore initially. Then, randomly generate a large number (~7000) of wavelength sets spanning 400–900 nm, using a uniform distribution to ensure broad coverage.
2. Dataset Construction For each candidate wavelength set, extract the corresponding bands from the hyperspectral data and construct training/testing datasets with labels.
3. MLP Training and Evaluation Train the MLP on each dataset and compute macro_avg as the performance metric. In the first iteration, only the top 10% of the wavelength sets are retained as candidates.
4. Selection of Top-Performing Wavelength Sets Cluster the candidate sets into 20 groups using k-means and evaluate each cluster’s average macro_avg. The top 50% of clusters (by average) plus clusters containing any wavelength set with an individually high macro_avg are kept as “seed” clusters. This step is inspired by the principle of successive halving, which focuses resources on promising subsets.
5. Generating the Next Generation of Wavelength Sets For each of the 20 seed clusters, randomly perturb each wavelength by a small offset. Specifically, we add random noise drawn from three different scales (0.1, 0.3, and 0.6 times the mean band spacing), producing a total of 720 new wavelength sets as the next generation. This ensures that the search explores the vicinity of each seed set with a controlled level of spread.

Iteration: Steps 2–5 are repeated until the wavelength sets converge. Typically, each iteration takes about 4 h, and convergence is decided once changes in the top 50% clusters become negligible.

**Figure 11:**
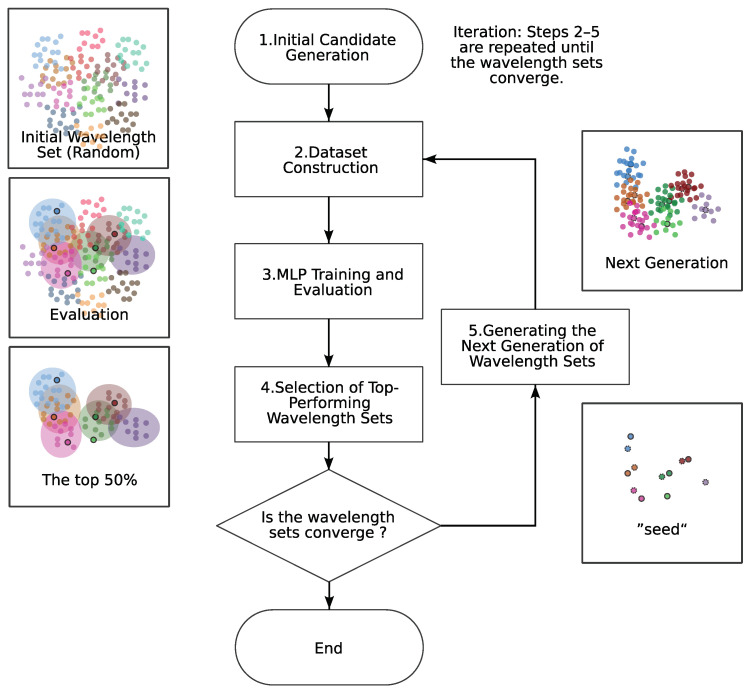
Flowchart of optimal wavelength set exploration (Steps 1–5 in the text). Different colored dots represent groups obtained via k-means clustering of candidate wavelength sets (for illustrative purposes only). See Section 4.3.2 for details.

**Figure 12:**
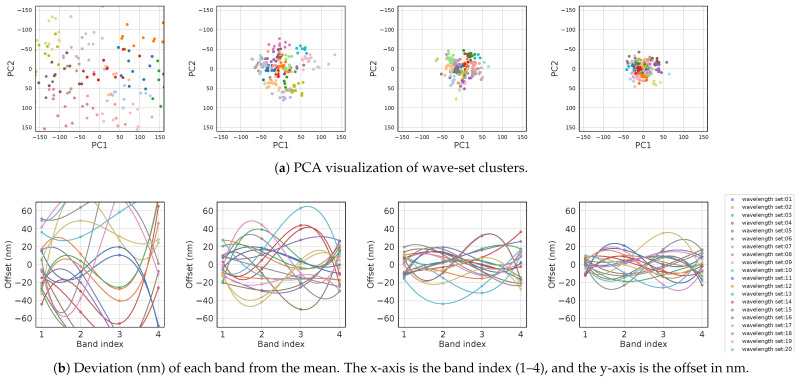
Example of convergence for the 4-band optimal wavelength set search (from **left** to **right**: initial state, iteration 1, iteration 2, iteration 5). (**a**) Principal component analysis (PCA) visualization of wave-set clusters. (**b**) Deviation (nm) of each band from the mean. The x-axis is the band index (1–4), and the y-axis is the offset in nm. Different colored lines indicate groups obtained via k-means clustering.

Figure 11 depicts this OWS search process. In Figure 12a, for the 4-band case, PCA visualization shows the clusters with color coding. In Figure 12b, we plot how each band deviates (in nanometers) from its initial average. Over time, the wavelengths converge to specific ranges. The figure confirms that while the initial wave sets are scattered widely, the search converges to higher-performing sets in subsequent loops. One loop took about 4 h. The results were accepted as convergence when changes in the top 50% of clusters became negligible, typically after four to six loops. We verified reproducibility by running the entire search four times, each converging to nearly the same wavelength sets.

Table 6 summarizes the final OWS results and evaluation metrics for 4-, 5-, and 3-band searches.

**Figure 13:**
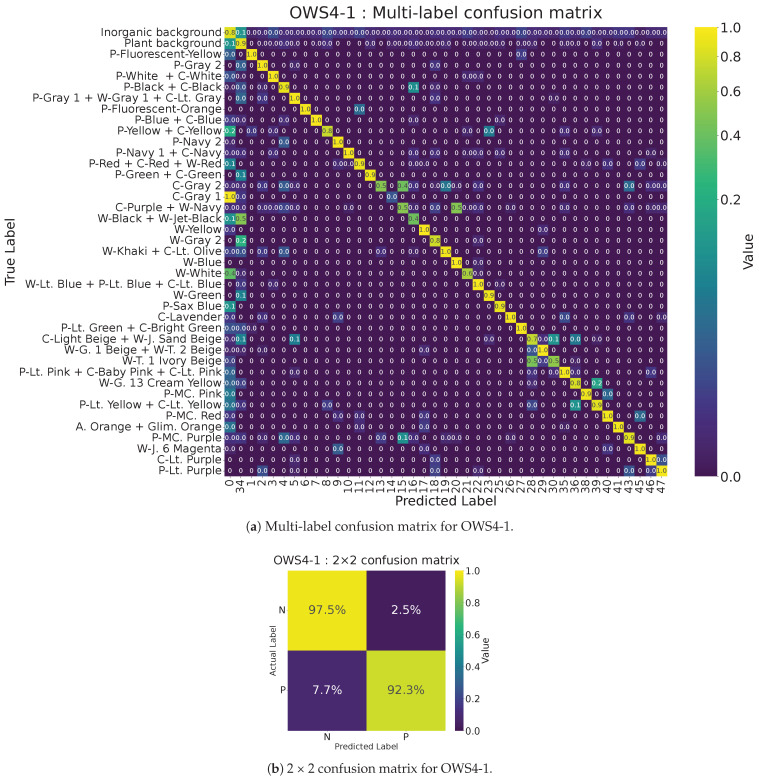
(**a**) Multi-label confusion matrix for optimal wavelength set with 4 bands (OWS4-1). (**b**) 2 × 2 confusion matrix for OWS4-1.

- 4-band search: Two solutions emerged, called OWS-4-1 and OWS-4-2. OWS4-1 = [453, 556, 668, 708 nm] and OWS4-2 = [446, 567, 647, 716 nm]. Both achieved accuracy of 0.95, precision of 0.97, recall of 0.93, and F1-score of 0.95, comparable to the results for the 12-band model. This is a significant reduction in the band count. Figure 13 shows (a) the multi-label confusion matrix and (b) the 2 × 2 version for OWS4-1.
- 5-band search: Two solutions also emerged: OWS5-1 = [444, 556, 623, 652, 709 nm] and OWS5-2 = [445, 548, 562, 675, 729 nm]. Both achieved accuracy of 0.95, precision of 0.97, recall of 0.93, and F1-score of 0.95.
- 3-band search: One solution, OWS3-1 = [445, 581, 713 nm], gave accuracy of 0.93, precision of 0.95, recall of 0.93, and F1-score of 0.94. This is lower than the results of the 4- or 5-band models.

**Figure 14:**
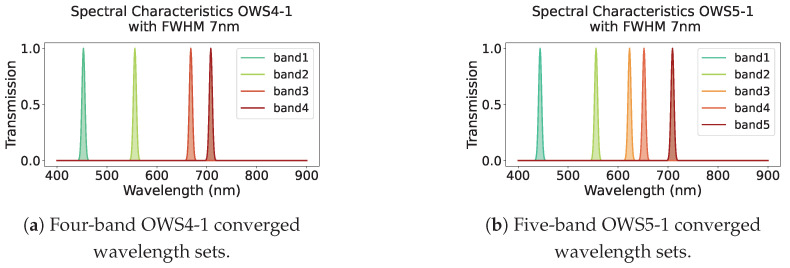

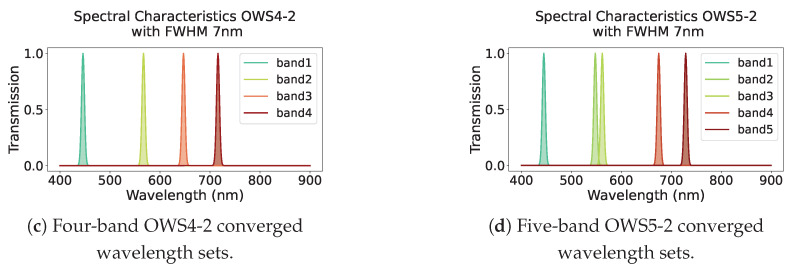
Passbands of the 4- and 5-band optimal wavelength sets (OWS). (**a**) OWS4-1. (**b**) OWS5-1. (**c**) OWS4-2. (**d**) OWS5-2.

Figure 14 graphically shows the passbands for the 4- and 5-band OWS solutions, revealing that the first and second wavelengths are similar, while the third and beyond differ. From these results, 4-band OWS can maintain performance comparable to a 12-band configuration despite fewer bands.

**Figure 15:**
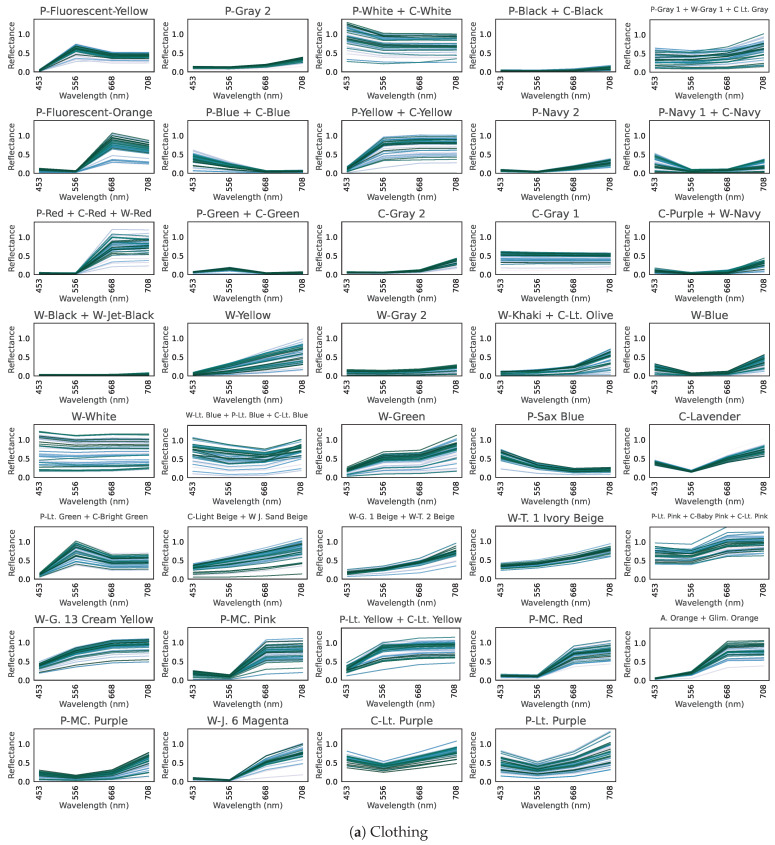

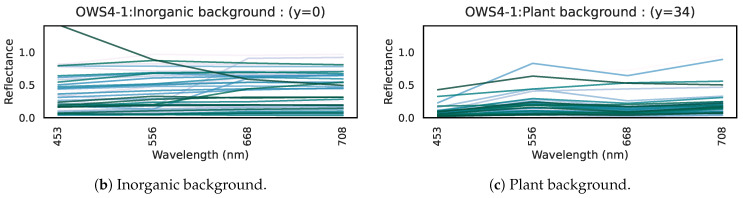
Multispectral reflectance at the four wavelengths of the optimal wavelength set (OWS4-1: 453, 556, 668, 708 nm) for (**a**) clothing, (**b**) inorganic background, and (**c**) plant background.

Figure 15 plots the multispectral reflectance for only these four OWS4-1 bands for examples of (a) clothing, (b) inorganic material, and (c) plants. Note the comparability to Figure 3, which shows the hyperspectral data. Despite having only four bands, the model attains the performance described above. The roles of these four bands are discussed in Section 6.

#### 4.3.3. Evaluation of Robustness of the Wavelength Sets

Shifts in the center wavelength or increases in the passband width can degrade detection performance. If performance degrades significantly, the required spectral-filter specifications may be too strict, complicating camera manufacturing. Hence, we intentionally varied the OWS4-1 and OWS5-1 sets to see how much performance would drop. We applied two types of variation:

1. Center-Wavelength Shift
  We shifted each wavelength individually. (We did not combine multiple simultaneous shifts due to the exponential growth in combinations.)
2. Broadening of Passband
  We simultaneously enlarged the passband width for all bands.

**Figure 16:**
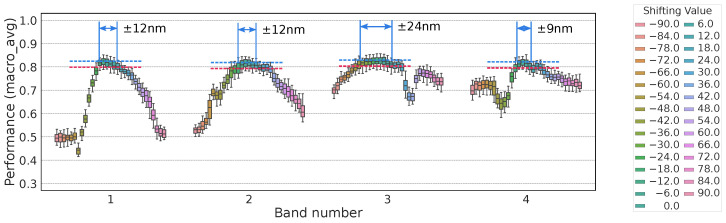
Performance variation when shifting the center wavelength of each of the 4 bands.

**Figure 17:**
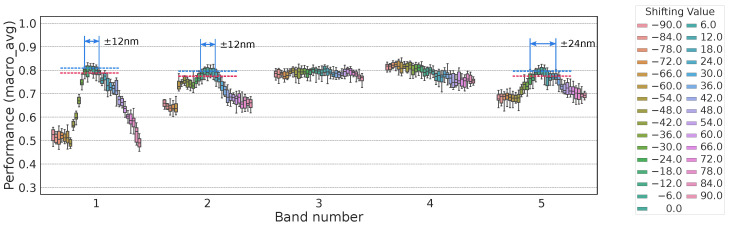
Performance variation for the 5-band set under center-wavelength shifts. Shifting the 3rd or 4th band alone had minimal impact (see text).

Figure 16 and Figure 17 show how macro_avg changed under these perturbations. As a rough guideline, we searched for the shift at which macro_avg dropped by 0.02.

- For OWS4-1, macro_avg fell by 0.02 when the center wavelength shift reached 12, 12, 24, and 9 nm for each band or when the passband was widened by 24 nm.
- For OWS5-1, the center-wavelength shift thresholds were ~12, 12, and 24 nm for the 1st, 2nd, and 5th bands, respectively, while shifting the 3rd or 4th band alone had little effect on macro_avg.

This implies that if the 4th band is present, the 3rd band is somewhat redundant (or vice versa). In other words, five bands may be more than necessary, and four bands could suffice. Overall, these results show that single-band shifts of about 10 nm are tolerable.

**Figure 18:**
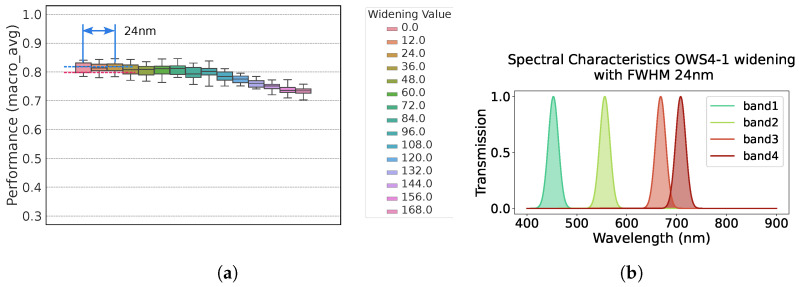
Performance variation when simultaneously widening the passband of all 4 bands. (**a**) performance of broadening passbands. (**b**) broadened passbands at 0.02 lower performance.

As for broadening passband widths, we examined only OWS4-1 (Figure 18a). Widening the passband by 24 nm lowered macro_avg by 0.02. Figure 18b also shows the resultant passband characteristics.

#### 4.3.4. Performance of Other Wavelength Sets

We tested several alternatives, such as 4- or 5-band cameras designed for agriculture [29,30,31], or sets of 12 bands in the visible range or in the near-infrared range. None achieved performance comparable to the four OWSs (OWS4-1, OWS4-2, OWS5-1, OWS5-2) listed in Table 6.

This indicates the importance of carefully selecting wavelengths for distinguishing clothing from the background and incorporating both visible and near-infrared regions.

In fact, alternative configurations—such as agricultural 4- or 5-band systems, 12-band setups in the visible range, or 12-band setups in the near-infrared range—performed worse (Table 7). This clearly demonstrates that the combination of visible and NIR wavelengths is key.

#### 4.3.5. MLP Inference Speed and Memory Usage

We measured the inference speed and memory usage of the clothing MLP (4-16-16-49), and compared them with those of YOLOv5 (s, m, x), Faster R-CNN, and EfficientDet [3,32,33]. These serve as representative spatial-pattern-based object detectors that are thoroughly studied in the research community, with well-documented reproducibility for measuring inference time and memory use. YOLOv5 provides multiple model sizes, so we evaluated three different ones to examine speed versus performance.

By comparing these methods, we aimed to clarify the advantages of the MLP. Note that we used the default hyperparameters and weights recommended by each official repository (YOLOv5, Faster R-CNN, and EfficientDet). Our MLP had fixed hidden layers of 16 × 2 units. We ran all tests on an NVIDIA TITAN V GPU.

**Figure 19:**
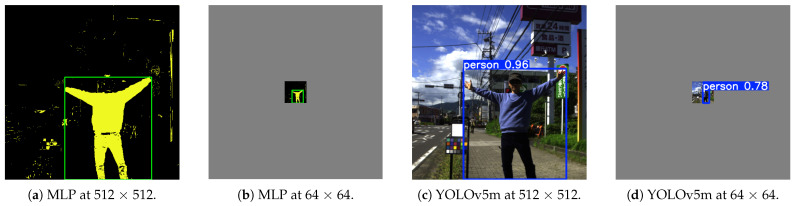
Example inference results at resolutions (**a**) multi-layer perceptron (MLP) at 512 × 512 pixels, (**b**) MLP at 64 × 64 pixels, and (**c**) YOLOv5m at 512 × 512 pixels, (**d**) YOLOv5m at 64 × 64 pixels. The MLP detects the boundary of clothing pixels and draws bounding boxes. It uses only spectral information from each pixel to decide whether it is clothing.

We used images of three different sizes (512 × 512, 128 × 128, and 64 × 64 pixels) as inputs and measured the inference speed for each model (Figure 19). To reconstruct the MLP results, we applied the pixel-level predictions in 2D. Spatial-pattern-based methods often lose performance when a person’s size becomes small. By contrast, because our MLP determines each pixel based on spectral data alone, its performance is less affected by lower image resolution.

**Figure 20:**
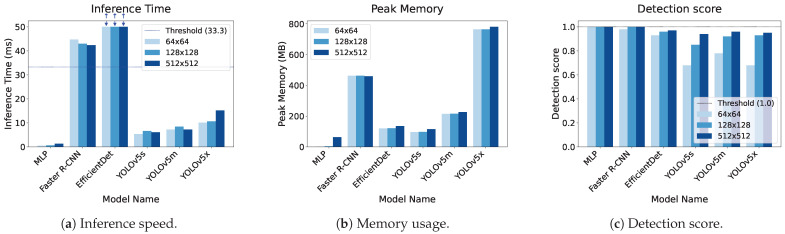
Inference speed, memory usage, and detection score. (**a**) Inference speed. The multi-layer perceptron (MLP) stays well below the 33 ms real-time threshold. (**b**) Memory usage. (**c**) Detection score.

Deep-learning models like YOLO are undoubtedly fast on supported hardware, but they may be less suitable for low-resource or embedded environments. Additionally, spectral methods can complement bounding-box approaches in cases of partial occlusions. Table 8 lists the speed, memory usage, and detection performance for a particular test image. Our MLP used only 63.0 MB for a 512 × 512 pixel image input and 1.02 MB for a 64 × 64 input, achieving inference times of 1.3 ms (512 × 512) to 0.5 ms (64 × 64). (Figure 20 shows the inference speed, memory usage, and detection score, respectively). Thus, the MLP is fast, memory-efficient, and robust to changes in spatial resolution.

Because our hyperspectral camera is intended for still images, we did not measure the frames per second (FPS) for a video stream. As shown in Table 8, the MLP processing (inference) requires only 1.3 ms per 512 × 512 pixel image, and the bounding box processing takes less than 1.0 ms, resulting in a total processing time of approximately 2.3 ms per image. YOLOv5m or YOLOv5s exceed 5 ms. Typically, real-time processing at 30 FPS is achievable if the processing time for each frame is under 33 ms. Therefore, MLP-based inference could theoretically achieve 30 FPS, although the duration of camera data transfer remains uncertain and is left for future work.

All spatial-pattern-based detectors consumed more memory than our MLP. These results confirm that a method exploiting only spectral data can require fewer computational resources. We plan to extend these tests to a multispectral camera supporting the video mode in future work, incorporating sensor frame rates and transfer times to confirm overall real-time feasibility.

(Summary of Section 4)

From the above results:

- MLP classification performance can be better with about 12 bands than with all 167 bands.
- With an optimal choice of bands, performance can be mostly maintained with as few as four bands.
- The method remains relatively robust to small shifts in wavelengths.

Hence, the 4-band OWS, with an F1-score of around 0.95, offers the best balance of detection accuracy, computational lightness, and practical feasibility. In the future, we plan to prototype a 4-band filter for a camera and evaluate its real-time performance outdoors under daylight conditions.

## 5. Experiments Using Actual Images

We investigated whether clothing could be accurately separated from the background in real images. Because the model performance can vary slightly depending on random factors in the training algorithm, we selected one MLP trained on OWS4-1 that yielded good results on sample images.

### 5.1. Validity of the 4-Band OWS Model

**Figure 21:**
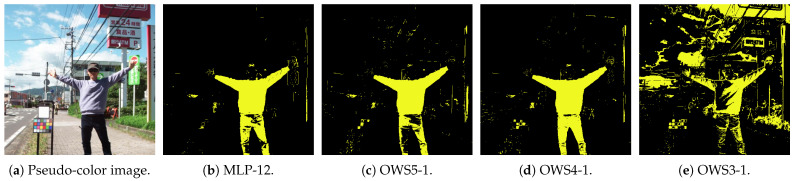
Examples of inference results from some wavelength set models with different band counts (12, 5, 4, and 3). Models with ≥4 bands perform well. (**a**) Pseudo-color (an artificially colored visualization) image. (**b**) 12-band multi-layer perceptron (MLP-12). (**c**) 5-band optimal wavelength set (OWS5-1) multi-layer perceptron. (**d**) 4-band OWS4-1 multi-layer perceptron. (**e**) 3-band OWS3-1 multi-layer perceptron.

Figure 21 compares inference results of models using different numbers of bands: MLP-12, OWS5-1, OWS4-1, and OWS3-1. Each result is a pixel-level classification reconstructed as a 2D image, where bright yellow indicates clothing and black indicates background. The clothing on the person was not included in the training data. The scene includes sky, ground, vegetation, signs, and other artificial objects.

Models with five (Figure 21c) or four (Figure 21d) bands maintained accuracy comparable to the 12-band model (Figure 21b), whereas the 3-band model (Figure 21e) gave more misclassification. Hence, the MLP with the 4-band OWS (Figure 21d) appears to be effective.

### 5.2. Generalization to Clothing Not Included in the Training

**Figure 22:**
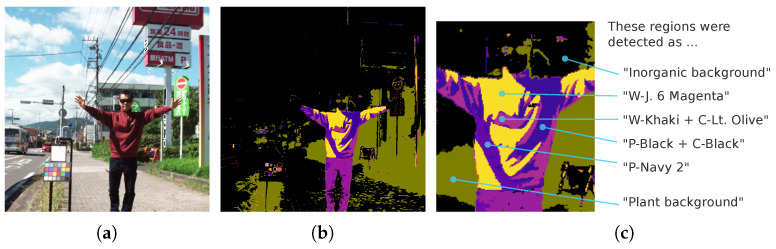
Example of a street scene containing a person with clothing not included in the training dataset. (**a**) Pseudo-color image. (**b**) 4-band optimal wavelength set (OWS4-1) multi-layer perceptron predictions. (**c**) Detailed labeling of the clothing region.

Figure 22a shows a pseudo-color image of a street scene containing a person wearing clothing for which the model was not trained. Figure 22b presents the pixel-level classification by the 4-band OWS MLP, color-coded by the predicted label. The MLP yields multi-label outputs: black for an inorganic background, dark green for a plant background, and other colors for clothing. The clothing color is determined by whichever label was assigned during training.

Although the jacket is composed of a single material and dye, the detection result merges multiple “clothing labels”. Figure 22c details which labels were recognized. A single garment can appear as multiple clusters of labeled regions—some illuminated by direct sunlight, others in shadow—yet they all belong to “clothing”. Furthermore, even if the inference yields different clothing labels, post-processing consolidates them into a single clothing region.

**Figure 23:**
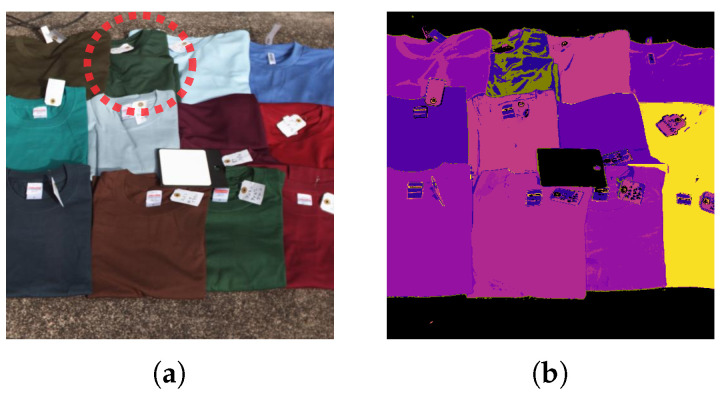
Samples of detected clothing not included in the training dataset. A single case misidentified as “plant” (red circle). (**a**) Pseudo-color image. (**b**) 4-band optimal wavelength set (OWS4-1) multi-layer perceptron predictions.

Figure 23 shows various samples of clothing not included in the training. In each case, the clothing was classified under at least one of the learned clothing labels (except for a single case misidentified as “plant”).

### 5.3. Analysis of Objects That Are Difficult to Detect

**Figure 24:**
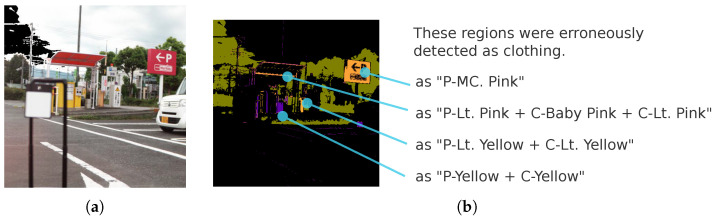
Example of a street scene containing background objects likely to be misclassified as clothing. (**a**) A scene including misclassified red and yellow objects. (**b**) Multi-label predictions by the 4-band optimal wavelength set (OWS4-1) multi-layer perceptron.

**Figure 25:**
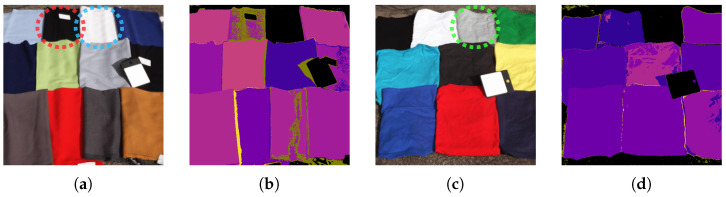
White (blue circle) or black (red circle) wool garments can be missed. (**a**) Pseudo-color image. (**b**) Multi-label predictions by the 4-band optimal wavelength set (OWS4-1) multi-layer perceptron. Gray cotton (green circle) garments can be missed. (**c**) Pseudo-color image. (**d**) Multi-label predictions by the 4-band optimal wavelength set (OWS4-1) multi-layer perceptron.

**Figure 26:**
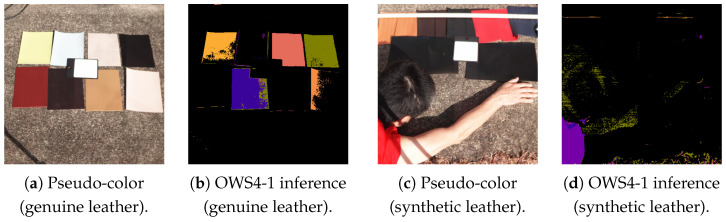
Examples of clothing materials deviating from this study’s “clothing hypothesis” (genuine leather and synthetic leather). (**a**) Pseudo-color image (genuine leather). (**b**) 4-band optimal wavelength set (OWS4-1) multi-layer perceptron inference (genuine leather). (**c**) Pseudo-color image (synthetic leather). (**d**) 4-band OWS4-1 multi-layer perceptron inference (synthetic leather).

Using a range of real-world scenes, we examined which objects might cause errors for this purely spectral-based method. Figure 24 shows an example street scene likely to produce false positives. Figure 25 illustrates clothing likely to be missed. Figure 26 shows clothing materials (genuine leather or synthetic leather) that deviate from this study’s “clothing hypothesis”.

In summary, the following cases are problematic:

- Any object perceived as red or yellow by the human eye and whose spectrum (extending into the near-infrared) closely matches that of clothing is prone to being misclassified as clothing, regardless of its material (Figure 24).
- White wool or black wool and Gray cotton garments may be missed (Figure 25).
- Clothing made of materials that deviate from the “fibers in the hypothesis” (Figure 26), for example, genuine or synthetic leather, may be missed.

**Figure 27:**
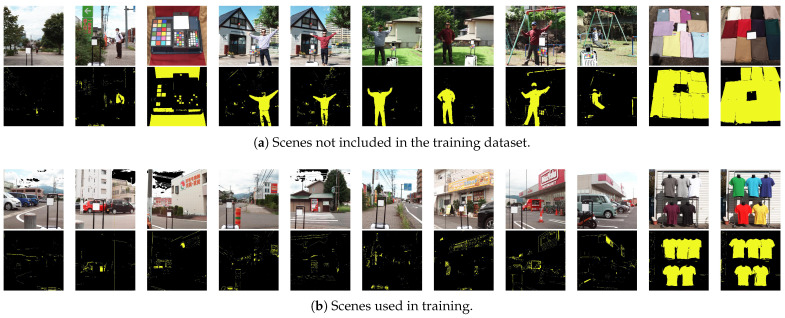
Overall inference examples from the 4-band optimal wavelength set (OWS4-1) multi-layer perceptron (top: pseudo-color (artificial color mapping) image; bottom: classification result with bright yellow for clothing and black for background). (**a**) Scenes not included in the training dataset. (**b**) Scenes used in training.

Finally, Figure 27 shows various scene examples with predictions by the 4-band OWS4-1 MLP. Panel (a) shows images not used in the training, and (b) consists of images used in training. Most unlearned samples are correctly detected as clothing.

## 6. Discussion

### 6.1. Effectiveness of Wavelength Selection

The results indicate that by selecting four wavelengths spanning the visible to near-infrared region, clothing detection is feasible to some extent. Using only four bands instead of the full spectrum simplifies the camera system and offers advantages for real-time capability.

Tests confirmed that using only the visible range or only the near-infrared was insufficient. Employing both visible and near-infrared wavelengths together proved critical. In other words, adding near-infrared to visible bands improved detection performance over visible-only approaches.

### 6.2. Effectiveness of Each Wavelength

**Figure 28:**
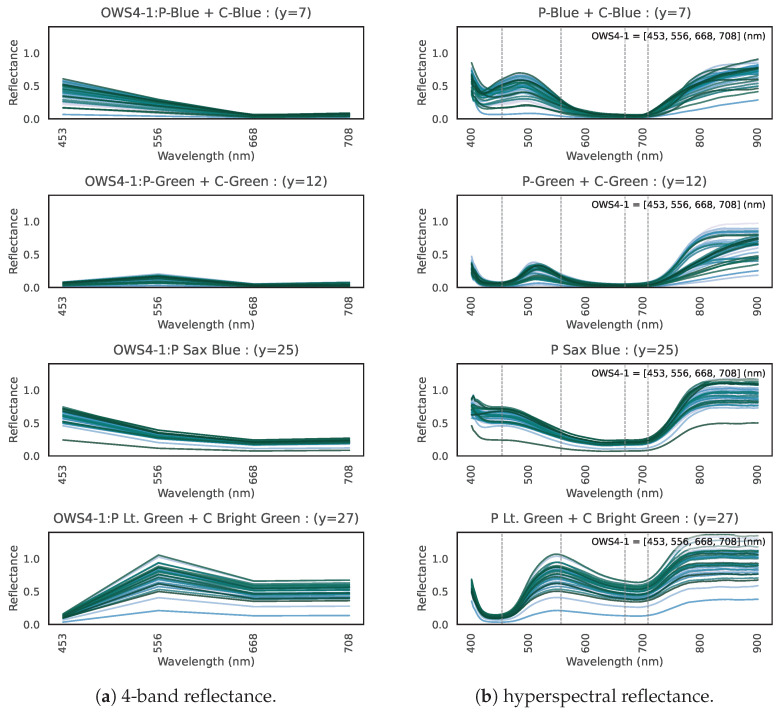
(**a**) 4-band reflectance and (**b**) hyperspectral reflectance for four types of green/blue garments.

**Figure 29:**
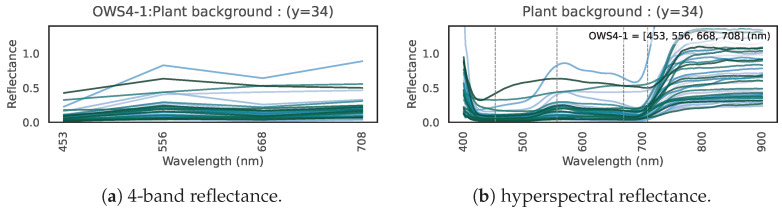
(**a**) 4-band and (**b**) hyperspectral reflectance for plants.

**Figure 30:**
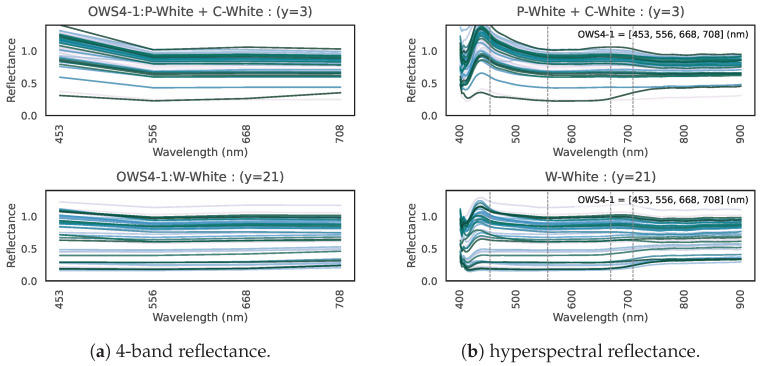
Reflectance at (**a**) 4 bands and (**b**) full hyperspectral data for white garments (polyester, cotton, and wool).

Why are these four specific wavelengths so effective? Here, we consider the physical and spectral background. We hypothesize the roles of the four bands in OWS4-1 as follows:

- Fourth Wavelength: Capturing “many garments”
  As shown in Figure 15, the majority of clothing (32 labels) exhibits high reflectance in the 4th wavelength. This band captures the high near-infrared reflectance of most clothing, consistent with our hypothesis that fibers/dyes often reflect well in the near infrared. In addition, combining this band with the second or third bands is helpful for identifying particular clothing colors.
- Combination of Second, Third, and Fourth Wavelengths: Distinguishing “green/blue clothing” from vegetation
  The second and third wavelengths alone do not appear to capture any single specific physical property. We infer that the reflectance pattern across these three bands (including the fourth) helps discriminate certain colors. For instance, the clothes labeled “P-Blue + C-Blue”, “P-Green + C-Green”, “P Sax Blue”, and “P Lt. Green + C Bright Green” have low reflectance at the 4th band. Figure 28a shows their 4-band reflectance, all satisfying 2nd band > 3rd band = 4th band. However, the hyperspectral curves in Figure 28b reveal that these green/blue garment curves do rise sharply beyond 708 nm. Because the 4th band (708 nm) is slightly short of the near-infrared region where reflection spikes, these garments appear to have lower reflectance at that band.
  However, vegetation that looks similarly green to the human eye shows 2nd band > 3rd band < 4th band, as indicated by Figure 29. Comparison of Figure 28a,b indicates that vegetation transitions to high near-infrared reflectance at a slightly shorter wavelength than green clothing. Hence, the 2nd, 3rd, and 4th wavelengths together capture this difference in the onset of near-infrared reflectance, allowing the model to distinguish green clothing from plants.
- First Wavelength: Capturing “white clothing”
  In Figure 30, we show (a) the 4-band reflectance and (b) the hyperspectral reflectance of white polyester/cotton garments (“P-White + C-White”). Their reflectance is very high (above 1.0) at the first band, likely due to fluorescent brighteners used in white fabric dyes [34,35,36]. Hence, the first wavelength is effective for capturing the high reflectance of P + C whites. White wool (“W-White”) may likewise incorporate fluorescent brighteners, as evidenced by a peak at approximately 440 nm in the 167-band data. However, the peak wavelength of these brighteners (~440 nm) inferred from the spectral curves does not fully coincide with the first wavelength (453 nm). This discrepancy suggests that the convergence approach employed in this study will benefit from further refinement.

### 6.3. Limitations of Spectral-Only Detection: Hypothesis, Material, Color Similarities, and Illumination Variations

- Counterexamples to the Hypothesis: Uncolored wool or gray cotton that do not increase near-infrared reflectance is easily missed. Nude bodies are not considered.
- Material Constraints: Leather or synthetic leather that do not reflect well in the near-infrared cannot be detected. Military camouflage clothing [37] or materials that are difficult to see even with the naked eye are beyond the scope of this method.
- Nighttime or Low-Light Environments: Our experiments assume outdoor daytime conditions. Nighttime use would require external infrared illumination, and the drastic changes in spectral distributions under different lighting conditions preclude direct application of our approach.
- Red or Yellow Background: If these colors exhibit spectral properties similar to those of clothing, false positives may occur.
- Illumination Spectral Variations and Transparent Materials: Detection performance may degrade under varying illumination conditions (e.g., direct sunlight versus shadow) due to significant changes in the spectral distribution. Moreover, imaging through transparent materials (e.g., glass) can further compromise detection accuracy because of spectral transmittance and optical effects. It should be noted that performance evaluation under these conditions has not yet been conducted and remains an important topic for future research.

Possible countermeasures include (1) collecting extra training data emphasizing these challenging garments or (2) introducing a hybrid method that combines spectral data with spatial pattern features such as texture or shape. However, adding more challenging clothing might also increase false positives for similar backgrounds. Furthermore, incorporating spatial pattern recognition can increase the computational demand, undermining the speed advantages of a purely spectral approach.

Hence, we consider it reasonable to “give up” in cases where the material does not satisfy the “fiber high near-infrared reflectance” assumption or where background objects share very similar spectra. In this research, we deliberately focused on the scenario of daytime outdoor people wearing normal fiber garments, which yields high recall within that domain.

To our knowledge, there are no clear statistical data confirming that most people in public areas wear textile garments, but in urban environments, this is generally true. Therefore, treating clothing detection as an approximation of human detection is likely to have sufficient merit.

### 6.4. Prospects for MLP Model Generalization

The dataset of hyperspectral images used in this study was limited, covering only daytime outdoor environments and a modest number of test images. Nighttime or highly dynamic situations have not been evaluated. Even so, the real-image results in Section 5 suggest some degree of generalization: the model detected clothing that was not part of the training.

Going forward, we must clarify the range of scenarios for which the “clothing hypothesis” is valid. A much larger dataset spanning various locations, times, weather conditions, and urban/rural settings will be needed to further assess the generality of clothing detection. With sufficient data from large-scale experiments, we can gain deeper insight into how broadly the method applies.

Because a still-image hyperspectral camera makes large-scale data collection difficult, we plan to use a smaller, video-capable spectral camera to capture diverse scenes and subjects continuously, thus enriching the training data.

### 6.5. Outlook for a Real-Time Camera System

The MLP approach offers fast, memory-efficient inference, suitable for IoT devices or compact cameras. Using only four bands reduces the complexity compared to high-end systems with many bands. From the robustness evaluation in Section 4.3.3, we can see that moderate variations in the center wavelength or passband width are tolerable, making it feasible to use off-the-shelf filters.

Small multispectral cameras with selectable wavelengths, such as polarization-based multispectral cameras [38] or multi-lens TOMBO cameras [39,40,41], can potentially be adapted to create a 4-band camera for human detection. In outdoor settings, however, illumination can vary by time and place, so we must address how to track or adapt to changing lighting conditions.

We plan to move forward with prototyping. Adapting to nighttime or indoor settings through infrared illumination or an improved sensor signal-to-noise ratio is a future challenge.

## 7. Conclusions

In this study, we proposed a color-based multispectral approach that uses only four selected wavelengths (453, 556, 668, and 708 nm) to detect clothing as a proxy for human detection under daytime conditions. The method emphasizes a reduced computational load by focusing on pixel-wise spectral reflectance rather than deep learning or extensive image segmentation. Our main findings and limitations can be summarized as follows:

1. High Accuracy with Fewer Wavelengths
  By carefully selecting four wavelengths in the visible to near-infrared range, we achieved a stable classification performance for clothing, reaching an F1-score around 0.95. This demonstrates that broad spectral coverage is not always necessary; a few well-chosen bands can suffice for robust detection.
2. Real-Time Suitability and Simple Configuration
  The pixel-based classification approach avoids large-scale deep learning models and maintains computational lightness, making it amenable to real-time processing. A multispectral camera with only four bands could thus support efficient human detection in embedded or resource-limited systems.
3. Limitations in Spectral-Only Detection
  Objects with visually similar spectral signatures (e.g., certain red or yellow surfaces, white wool versus gray cotton) can lead to misclassification or false positives. Furthermore, the current approach is restricted to daytime illumination and common fabrics (cotton, polyester, wool), potentially reducing its applicability to nighttime scenarios or less typical materials.
4. Need for Combined Approaches
  While purely spectral methods can be fast, they may struggle in scenes with high spectral overlap or partial occlusions. Integrating spatial pattern recognition or extending to additional wavelengths may be needed to improve robustness without undermining the simplicity of the proposed system.

Overall, our findings indicate that multispectral imaging, relying on a small set of well-chosen bands, can effectively distinguish clothing under real-world conditions, while imposing relatively low computational demands. Future work will extend this approach to larger-scale video datasets, more diverse fabrics, and potentially nighttime or indoor environments. By combining spectral cues with spatial or temporal analysis, we anticipate further gains in accuracy and robustness, paving the way for real-time human detection in security, autonomous driving, and search-and-rescue applications.

## Figures and Tables

**Table 1:** List of 41 labels (label name, index, and R-channel intensity in the labeled image).

Label Name ^1^	Index	R-Channel Intensity
Inorganic background	0	0
Plant background	34	255
P-Fluorescent-Yellow	1	53
P-Gray 2	2	245
P-White + C-White	3	67
P-Black + C-Black	4	204
P-Gray 1 + W-Gray 1 + C-Lt. Gray	5	145
P-Fluorescent-Orange	6	135
P-Blue + C-Blue	7	219
P-Yellow + C-Yellow	8	127
P-Navy 2	9	187
P-Navy 1 + C-Navy	10	72
P-Red + C-Red + W-Red	11	178
P-Green + C-Green	12	248
C-Gray 2	13	244
C-Gray 1	14	189
C-Purple + W-Navy	15	64
W-Black + W-Jet-Black	16	197
W-Yellow	17	128
W-Gray 2	18	65
W-Khaki + C-Lt. Olive	19	171
W-Blue	20	241
W-White	21	153
W-Lt. Blue + P-Lt. Blue + C-Lt. Blue	22	240
W-Green	23	188
P-Sax Blue	25	246
C-Lavender	26	236
P-Lt. Green + C-Bright Green	27	252
C-Light Beige + W-J. Sand Beige	28	233
W-G. 1 Beige + W-T. 2 Beige	29	227
W-T. 1 Ivory Beige	30	214
P-Lt. Pink + C-Baby Pink + C-Lt. Pink	35	251
W-G. 13 Cream Yellow	36	206
P-MC. Pink	38	253
P-Lt. Yellow + C-Lt. Yellow	39	243
P-MC. Red	40	239
A. Orange + Glim. Orange	41	203
P-MC. Purple	43	247
W-J. 6 Magenta	45	221
C-Lt. Purple	46	232
P-Lt. Purple	47	249

^1^ Polyester is abbreviated as “P-”. Cotton is abbreviated as “C-”. Wool is abbreviated as “W-”. Toray is abbreviated as “T.”. Gabardine is abbreviated as “G.”. Mixed color is abbreviated as “MC.”. Josette is abbreviated as “J.”. Athletic is abbreviated as “A.”. Glimmer is abbreviated as “Glim.”. Grayish is abbreviated as “G.”. Light is abbreviated as “Lt.”.

**Table 2:** Comparison of five machine learning approaches: radial basis function support vector machine (RBF-SVM), random forest, gradient boosting, adaptive boosting, and multi-layer perceptron (MLP).

Model	Accuracy Score	Precision	Recall	F1 Score
RBF-SVM	0.922	0.88	0.525	0.658
Random Forest	0.846	0.471	0.592	0.524
Gradient Boosting	0.904	0.787	0.452	0.574
Adaptive Boosting	0.901	0.776	0.437	0.559
MLP	0.921	0.815	0.586	0.682

**Table 3:** Results of preliminary experiments on the multi-layer perceptron (MLP) hidden units (6/10/16/32/64). Performance plateaued with 16 units × 2 layers.

Model	Accuracy	Precision	Recall	F1 Score	Band1 (nm)	Band2 (nm)	Band3 (nm)	Band4 (nm)	Band5 (nm)	...	Band20 (nm)
MLP: 20-6-6-49	0.884	0.94	0.837	0.885	413	438	463	488	513	...	888
MLP: 20-10-10-49	0.917	0.95	0.893	0.92	413	438	463	488	513		888
MLP: 20-16-16-49	0.928	0.965	0.899	0.931	413	438	463	488	513		888
MLP: 20-32-32-49	0.924	0.967	0.889	0.927	413	438	463	488	513		888
MLP: 20-64-64-49	0.935	0.97	0.908	0.938	413	438	463	488	513		888

**Table 4:** Accuracy/Precision/Recall/F1 Score for 167-band Multi-Layer Perceptron (MLP-167).

Model	Accuracy	Precision	Recall	F1 Score	Band1 (nm)	Band2 (nm)	Band3 (nm)	Band4 (nm)	Band5 (nm)	...	Band167 (nm)
MLP-167	0.934	0.98	0.897	0.936	400	403	406	409	412	...	900

**Table 5:** Comparison of evaluation metrics for multi-layer perceptron (MLP) models with reduced dimensions by uniformly subsampling wavelengths or by principal component analysis (PCA).

Model	Accuracy	Precision	Recall	F1 Score
MLP-150 (400~900)	0.924	0.976	0.881	0.926
MLP-70 (400~900)	0.931	0.98	0.892	0.933
MLP-20 (400~900)	0.934	0.978	0.898	0.936
MLP-12 (400~900)	0.926	0.961	0.9	0.929
MLP-12 (430~770)	0.947	0.970	0.931	0.950
MLP-9 (420~800)	0.931	0.964	0.906	0.934
PCA-12	0.921	0.978	0.874	0.923
PCA-8	0.917	0.975	0.87	0.919
PCA-6	0.91	0.975	0.857	0.912

**Table 6:** Final optimal wavelength set (OWS) combinations and evaluation metrics for 4-, 5-, and 3-band searches.

Model	Accuracy	Precision	Recall	F1 Score	Band1 (nm)	Band2 (nm)	Band3 (nm)	Band4 (nm)	Band5 (nm)
OWS4-1	0.947	0.97	0.932	0.95	453	556	668	708	-
OWS4-2	0.946	0.968	0.932	0.949	446	567	647	716	-
OWS5-1	0.947	0.97	0.932	0.95	444	556	623	652	709
OWS5-2	0.943	0.969	0.924	0.946	445	548	562	675	729
OWS3-1	0.93	0.945	0.926	0.935	445	581	713	-	-

**Table 7:** Performance of Additional Wavelength Set Configurations: Agricultural-Use and Range-Limited (Visible-Only and Near-Infrared-Only) Examples.

Model	Accuracy	Precision	Recall	F1 Score	Band1 (nm)	Band2 (nm)	Band3 (nm)	Band4 (nm)	Band5 (nm)	...	Band12 (nm)
Parrot Sequoia	0.822	0.94	0.714	0.811	550	659	734	790	-	-	-
DJI P4	0.914	0.963	0.874	0.916	449	560	650	730	839	-	-
MicaSense RedEdge3	0.919	0.963	0.883	0.921	475	560	668	716	839	-	-
MLP-NIR12	0.859	0.951	0.778	0.855	651	673	694	716	738	...	889
MLP-VIS12	0.885	0.944	0.835	0.886	412	435	458	482	505	...	668

**Table 8:** Comparison of speed, memory, and detection score for a sample test image.

Model Name ^1^	Input Image Size	Peak Memory (MB)	Inference Time (ms)	Detection Score
MLP	64 × 64	1.02	0.5	1
MLP	128 × 128	3.97	0.6	1
MLP	512 × 512	63.04	1.3	1
Faster R-CNN	64 × 64	462.91	44.7	0.98
Faster R-CNN	128 × 128	463.05	43	1
Faster R-CNN	512 × 512	459.56	42.4	1
EfficientDet	64 × 64	119.65	184.7	0.93
EfficientDet	128 × 128	121.15	146.8	0.96
EfficientDet	512 × 512	134.77	145.4	0.97
YOLOv5s	64 × 64	96.5	5.3	0.68
YOLOv5s	128 × 128	96.9	6.6	0.85
YOLOv5s	512 × 512	114.9	6.1	0.94
YOLOv5m	64 × 64	214.57	7.2	0.78
YOLOv5m	128 × 128	215.07	8.4	0.92
YOLOv5m	512 × 512	225.74	7.2	0.96
YOLOv5x	64 × 64	763.85	10.1	0.68
YOLOv5x	128 × 128	764.58	10.6	0.93
YOLOv5x	512 × 512	780.48	15.2	0.95

^1^ Multi-layer perceptron (MLP).

## Data Availability

Due to corporate confidentiality and proprietary considerations, the datasets generated and/or analyzed during the current study are not publicly available. However, summary statistics and non-sensitive aggregated results are available from the corresponding author upon reasonable request.
